# Efficient electrodiagnosis of polyneuropathy—a targeted three-nerve conduction study protocol

**DOI:** 10.3389/fneur.2026.1862881

**Published:** 2026-07-28

**Authors:** Orit Mazza, Eyal Heled, Mohammad Khamaisi, Itay Cohen, Manuel Zwecker

**Affiliations:** 1 Department of Orthopedics, Loewenstein Rehabilitation Medical Center, Raanana, Israel; 2 Hospital Management, Edith Wolfson Medical Center, Holon, Israel; 3 Gray Faculty of Medical and Health Sciences, Tel Aviv University, Tel Aviv, Israel; 4 Department of Psychology, Ariel University, Ariel, Israel; 5 Department of Neurological Rehabilitation, Division of Rehabilitation, The Integrated Rehabilitation Hospital, Sheba Medical Center, Tel HaShomer, Israel; 6 Rutgers School of Public Health, Rutgers University, Piscataway, NJ, United States

**Keywords:** diagnosis, electrodiagnosis, electrophysiological studies, nerve conduction studies, polyneuropathy

## Abstract

**Introduction:**

Electrodiagnostic testing is central to the evaluation of polyneuropathy, yet substantial variability exists in nerve selection and testing protocols. This study aimed to identify the minimum nerve set that provides high sensitivity and overall diagnostic accuracy while retaining adequate specificity. Testing two motor and two sensory nerves was hypothesized to be diagnostically sufficient.

**Methods:**

We conducted a retrospective cross-sectional cohort study using a deidentified database from the electrophysiology laboratory at Sheba Medical Center. Patients diagnosed with polyneuropathy were compared with individuals who had normal nerve conduction studies. Groups were matched for age and sex, and patients with coexisting conditions such as mononeuropathies or polyradiculopathies were excluded. Associations between nerve conduction parameters and polyneuropathy were examined using chi-square analyses. Parameters with significant associations were entered into separate multivariable logistic regression models for sensory and motor nerves. ROC analyses evaluated individual nerves and progressively expanded nerve-testing protocols. Optimal sensitivity and specificity were determined.

**Results:**

Among 455 patients, 259 had polyneuropathy, yielding 91 matched patient–control pairs. Binary sensory abnormalities of the sural and ulnar nerves were the strongest and most informative discriminators. Motor abnormalities were most prominent in the tibial and ulnar nerves. The initial three-nerve protocol, comprising sural sensory, tibial motor, and ulnar sensory and motor studies, achieved an AUC of 0.916, with 80.5% sensitivity and 89.6% specificity. Adding contralateral ulnar sensory testing increased the AUC only marginally to 0.923 and reduced sensitivity to 76.8%.

**Conclusion:**

The three-nerve protocol provided a favorable balance between sensitivity and specificity. These findings support a stepwise approach beginning with unilateral sural, tibial, and ulnar studies, followed by contralateral testing when needed to increase diagnostic confidence.

## Introduction

Polyneuropathy (PNP) is a widespread condition impacting several peripheral nerves, typically in a length-dependent manner, resulting from factors like diabetes, toxins, metabolic disorders, and genetic predispositions (1). The diagnosis of large-fiber polyneuropathy is based on clinical suspicion supported by electrodiagnostic confirmation, as nerve conduction studies (NCS) provide objective evidence of large fiber dysfunction (2). PNP ranks among the most common neurological disorders, and electrophysiological evaluations for PNP constitute a significant part of the work conducted in Electromyography (EMG) laboratories. Electrophysiological assessment is a key tool for diagnosing peripheral neuropathy and, in specific subtypes such as acquired demyelinating inflammatory neuropathies, it provides valuable information that can guide prognosis and therapeutic decisions (3). Furthermore, it plays a crucial role in differential diagnosis, particularly in distinguishing PNP from mononeuropathies and radiculopathies that may present similar symptoms (4).

While NCS are always individualized based on clinical presentation and evolving findings during the study, PNP poses a unique challenge because its diffuse and length-dependent nature requires assessment of both sensory and motor nerves from different regions (5). Thus, evaluation is less restricted to a single protocol and instead relies on sampling across several nerves to capture the extent of involvement.

Earlier studies revealed wide variation in examination techniques, result interpretation, and diagnostic criteria across European centers. Collaboration and guideline development have since improved consistency. However, a major challenge in electrodiagnosing polyneuropathy remains: the lack of consensus on optimal nerve conduction methods, testing protocols, and the selection of nerves and muscles to assess. Current guidelines primarily address inflammatory subtypes, most notably AIDP (acute inflammatory demyelinating polyradiculoneuropathy) and CIDP (chronic inflammatory demyelinating polyradiculoneuropathy), as outlined in the European Academy of Neurology/Peripheral Nerve Society (EAN/PNS) recommendations. In contrast, evidence-based guidance for non-inflammatory neuropathies remains limited (6–8).

Key uncertainties remain regarding the number of nerves to test and the minimum findings required for diagnosis (9). Traditional protocols favor broad sampling, but recent studies support a more targeted approach, testing both sensory and motor nerves from upper and lower limbs. Most guidelines highlight the sural sensory and peroneal or tibial motor nerves for their sensitivity in detecting length-dependent neuropathies (10). Emerging evidence suggests that adding nerves such as the plantar nerves may improve early detection, particularly in distal symmetric polyneuropathy (DSP). These gaps underscore the need for updated, standardized, data-driven protocols (11).

The AANEM criteria for the electrodiagnosis of DSP recommend beginning with studies of the peroneal motor and sural sensory nerves. If both are normal, polyneuropathy can often be excluded without further testing (2). However, a prospective study of 313 patients found the tibial nerve to be more sensitive than the peroneal, while sural, dorsal sural, and medial plantar nerves also showed good sensitivity without side differences (12).

Machine learning has also been applied: one study classified diabetic sensorimotor PNP severity using an ensemble model with high accuracy (93%), sensitivity (92%), and specificity (98–99%). However, it relied only on two motor (median, peroneal) and two sensory (median, sural) nerves, and focused on F-wave, conduction velocity, and amplitude (13). Other parameters, such as distal latency and examination of other important nerves (ulnar and tibial nerves), were not included in the study (2, 14).

To address these limitations, the primary objective of this study was to evaluate the diagnostic contribution of individual sensory and motor NCS for identifying large-fiber PNP in routine electrodiagnostic practice. Specifically, we aimed to identify the nerves and electrophysiological parameters with the highest diagnostic value and to determine whether a focused, limited testing protocol could provide sufficient diagnostic confidence while minimizing patient discomfort and unnecessary examination burden. Because previous studies have not clearly defined the incremental value of bilateral testing, we also examined whether unilateral testing may be diagnostically sufficient and whether testing the contralateral side provides additional diagnostic confidence. It was hypothesized that the examination of two motor nerves and two sensory nerves would generally provide sufficient diagnostic certainty. In cases where uncertainty remains, testing an additional nerve may help exclude alternative diagnoses or strengthen the overall diagnostic conclusions. It was further hypothesized that bilateral testing would add confidence, although the incremental value of the second side would be relatively limited compared with the primary contribution of the first tested side.

## Materials and methods

### Cohort selection and study design

This retrospective cross-sectional cohort study used a de-identified electronic health record database from the electrophysiology unit, covering examinations of both inpatients and outpatients between November 2017 and February 2023 for adults aged 18 years or older. This manuscript was prepared in accordance with the Strengthening the Reporting of Observational Studies in Epidemiology (STROBE) Statement for cross-sectional studies. Electrophysiological diagnoses were coded using the International Classification of Diseases, 10th Edition (ICD-10). The study cohort consisted of patients with an ICD-10 code for polyneuropathy (G62.9), representing large-fiber polyneuropathies confirmed by electrodiagnostic evaluation. These included sensory, motor, and sensorimotor presentations with axonal, demyelinating, or mixed electrophysiological patterns. The comparison group included patients with normal electrodiagnostic findings (Z00.00). Patients with additional diagnoses, such as polyradiculopathy or other concomitant mononeuropathy, were excluded from the cohort (*n* = 82). To improve the accuracy of case identification based on ICD-10 codes, both the clinical reports and the coded diagnoses were independently reviewed by an electrodiagnostic physician and verified by another for consistency. The Institutional Ethics Committee granted ethical approval in accordance with the requirements for medical records research (SMC-0203-23).

The study followed the Knowledge Discovery in Databases (KDD) methodology, comprising three main stages (15). In the pre-processing stage, data were extracted from the database by SQL codes, cleaned to remove errors and handle missing values, and analyzed to select relevant variables. The analysis stage involved applying Chi-square tests and logistic regression. Finally, results were reviewed by experts and visualized for interpretation.

### Electrophysiological studies

**Figure 1:**
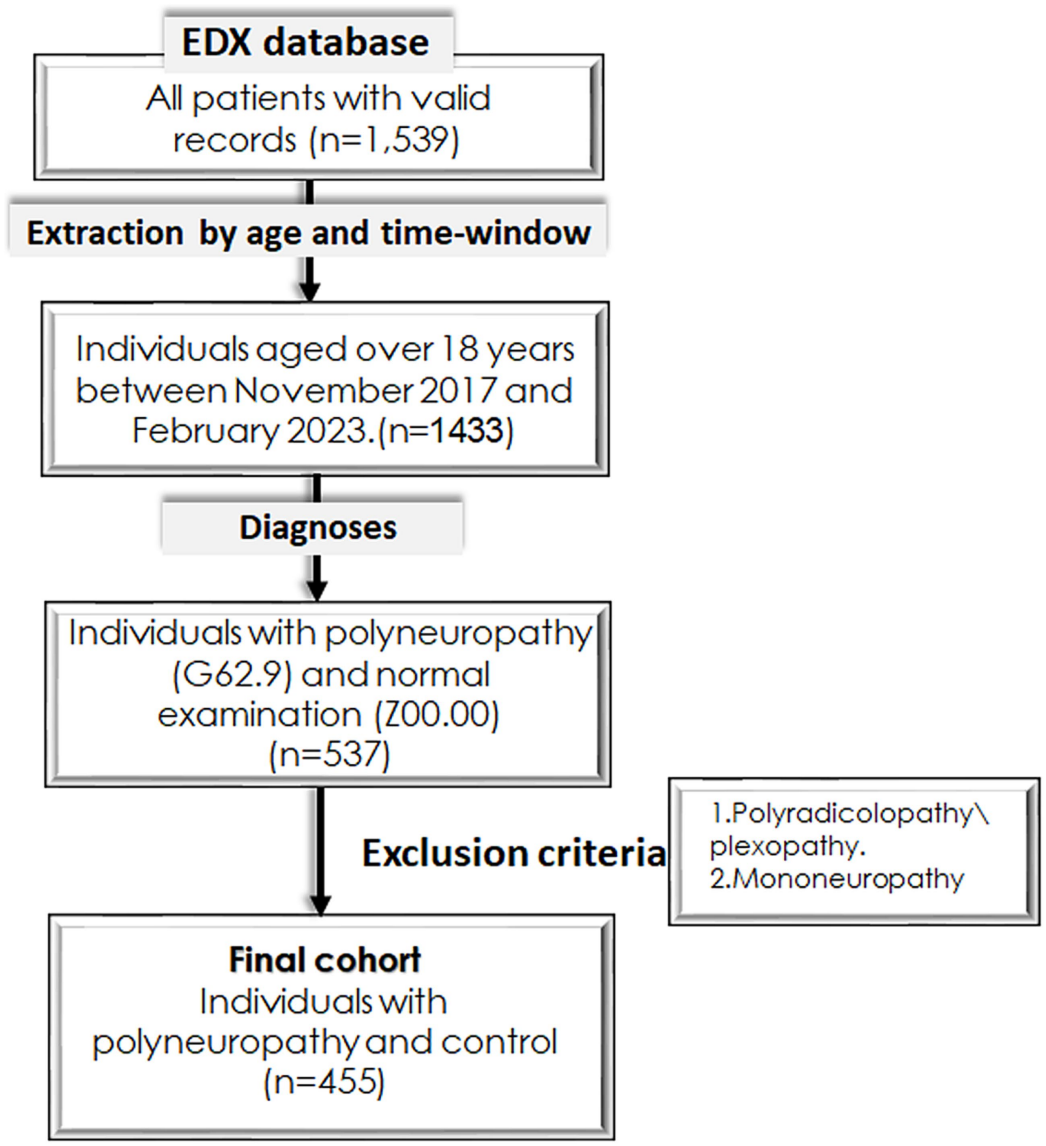
Cohort flowchart of patients with polyneuropathy and controls. Figure 1 presents the cohort flowchart, outlining the selection of individuals who underwent electrodiagnostic testing and the identification of those who met the inclusion criteria for either polyneuropathy or normal findings. It also shows the final classification of the study groups.

All electrodiagnostic examinations were performed at the laboratory by experienced electromyographers with at least 5 years of experience in electrodiagnostic medicine. The Nicolet VikingQuest two-channel EMG machine (Viking Software A/S, Odense, Denmark) was used for performing NCS. The parameters generated for each patient were automatically transferred to an Access database comprising seven main tables, linked through key identifier fields. The database contained anonymized information for 1,539 subjects, as shown in Figure 1, and included more than 100 clinical, anatomical, and electrodiagnostic parameters. The NCS protocol and the laboratory reference limits used to classify sensory and motor nerve conduction parameters as normal or abnormal are provided in Supplementary Table S1.

### Primary outcome

The primary outcome was defined as a binary variable indicating the presence or absence of polyneuropathy, based on NCS findings and corresponding ICD-10 codes (1 = polyneuropathy diagnosis, 0 = normal examination). The database contained records of 1,539 individuals who underwent electrodiagnostic testing. From this cohort, 455 individuals met the inclusion criteria of either confirmed polyneuropathy or normal findings. Among them, 259 patients were diagnosed with polyneuropathy. The cohort selection process is illustrated in Figure 1.

### The independent variables

The extracted data included demographic variables (age, gender, height) and NCS parameters. For motor NCS, the parameters included were onset latency, compound muscle action potential (CMAP) amplitude, and conduction velocity. For sensory NCS, the parameters included were peak latency and sensory nerve action potential (SNAP) amplitude. During the pre-processing stage, 20 sensory and motor nerves were screened for data completeness. Nerves with more than 50% missing values were excluded. The final set of nerves was selected following expert review by an electrodiagnostic specialist to assess the feasibility of including nerves with higher missing data rates. In clinical and electrodiagnostic datasets, the absence of a measurement is often not random. Whether an NCS is performed or recorded may depend on clinical judgment, disease severity, or prior findings. This mechanism is known as informative missingness, and several studies in medical decision-making and electronic health record (EHR) data have shown that the absence of testing can itself carry predictive information (16, 17). Therefore, two sets of parameters were generated: (1) binary variables indicating whether each nerve was examined (1 = tested, 0 = not tested) and (2) standardized NCS measures (latency, amplitude, and conduction velocity) for nerves with less than 50% missing data. Modeling “nerve tested” as a binary indicator alongside the actual measured NCS parameters allows for capturing two distinct types of information: the decision to test and the result of the test itself. This approach of incorporating the missingness mechanism can reduce bias and improve predictive performance when missingness is correlated with the outcome (17).

Specifically, this approach was intended to reduce the risk of bias from complete-case exclusion by retaining partially observed examinations, thereby reducing the loss of precision associated with discarding incomplete records (18). Therefore, retaining partially observed examinations and explicitly accounting for missingness patterns is preferable in retrospective clinical data, where the absence of a measurement may itself be informative (18). By allowing the model to use the testing pattern itself as an additional predictor when missingness was informative, this approach preserved clinically relevant information that would otherwise be lost if incompletely observed examinations were excluded (17, 19).

In this study, following the data extraction process, the examined nerves included the median nerve (sensory and motor), ulnar nerve (sensory and motor), and radial nerve (sensory and motor) in the upper limbs. In the lower limbs, the tibial, peroneal (superficial and deep branches), and sural nerves were analyzed. The superficial peroneal and radial nerves were included only in the set of binary parameters (tested vs. not tested) due to a high proportion of missing data, but were retained for their potential clinical relevance, as confirmed by expert review.

### Statistical analysis

Statistical analyses were conducted using R, version 4.4.2 (R Core Team, Vienna, Austria) (20), SPSS Statistics, version 27.0 (IBM Corp., Armonk, NY, United States) (21), and MedCalc Statistical Software (Version 23.6.1; MedCalc Software Ltd., Ostend, Belgium).

Age was assessed for normality using the Shapiro–Wilk test. Because age was not normally distributed, group comparisons were conducted using the Mann–Whitney U test implemented via the wilcox.test function in R. Gender was reported as counts and percentages (*n*, %) and compared between groups using the chi-squared (*χ*^2^) test. Missing electrophysiological data were handled using multiple imputation with Fully Conditional Specification (FCS) in SPSS. Five imputed datasets were generated using 10 iterations per imputation cycle. This approach was chosen to retain participants with partially missing data, thereby minimizing the loss of statistical power and potential bias associated with complete-case analyses (22). FCS was selected because it permits the specification of imputation models appropriate to the distribution of each incomplete variable, including logistic regression models for binary variables (23). Imputation was used to estimate missing measured values, whereas tested/not-tested indicators were used to model the missingness pattern itself. These two components, therefore, addressed complementary rather than contradictory sources of information.

Because significant between group differences were identified in age and gender, a matched dataset was created using the matchit function from the MatchIt package in R (24). Group differences across NCS parameters were assessed using chi-squared tests, and logistic regression analyses were conducted separately for sensory and motor nerves, including only variables significant in the chi-squared tests (*α* = 0.05).

To evaluate whether the available sample size was sufficient to detect the observed diagnostic accuracy of the proposed protocol, a sample-size adequacy analysis based on the receiver operating characteristic (ROC) was performed using MedCalc. The analysis assumed a null hypothesis Area ander the Curve (AUC) of 0.50, the observed AUC of the proposed protocol (AUC = 0.916), a two-sided significance level of *α* = 0.05, and 91 participants in each group.

To further evaluate the diagnostic utility of the NCS, ROC curve analyses were conducted. First, each sensory and motor nerve was evaluated using its binary classification as normal (0) or abnormal (1) according to the electrophysiological criteria employed in the study.

Second, progressively expanded diagnostic protocols were evaluated. Nerves included in these analyses were selected based on the results of the pooled multiple-imputation logistic regression analyses. Specifically, nerves demonstrating independent associations with polyneuropathy were entered sequentially into logistic regression models, reflecting increasingly comprehensive diagnostic protocols. Predicted probabilities derived from each logistic regression model were used as test variables in ROC analyses. Sensitivity, specificity, and the AUC were calculated to determine the diagnostic accuracy associated with the addition of successive nerve conduction studies.

## Results

Before matching, the extracted dataset included 455 individuals: 259 patients with PNP and 196 controls with normal NCSs. Patients with PNP were significantly older than controls (mean age 62.3 ± 16.2 years vs. 38.2 ± 15.5 years, *p* < 0.001) and more frequently male (68.7% vs. 42.9%, *p* < 0.001). After matching age and gender, the cohort comprised 182 subjects (91 in each group). No significant differences remained between the PNP and control groups (mean age 48.9 ± 17.5 years vs. 46.5 ± 16.1 years, *p* > 0.05; males 53.8% vs. 52.7%, *p* > 0.05). The *post hoc* ROC-based power analysis showed that the final sample size was adequate. The cohort of 91 patients with PNP and 91 matched controls provided estimated power greater than 0.99 for detecting the observed diagnostic accuracy of the proposed protocol.

**Figure 2:**
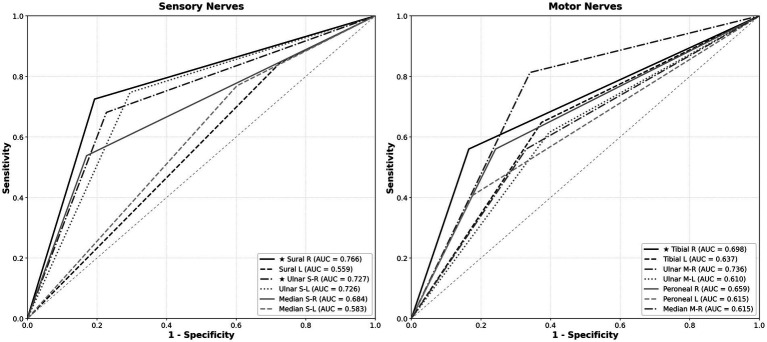
ROC Curves of Individual Sensory and Motor Nerves for Polyneuropathy Detection. ROC curves demonstrate the discriminative performance of individual sensory nerves and motor nerves for detecting polyneuropathy. Each nerve was classified as normal or abnormal according to the reference limits used in the study. Stars indicate nerves included in the proposed three-nerve protocol: right sural sensory, right tibial motor, and right ulnar sensory and motor nerves.

Demographic characteristics before and after matching are presented in Appendix Tables S2, S3, respectively. In addition, Appendix Figures S1, S2 represent the normality of age before and after matching, respectively. ROC analyses of individual nerves demonstrated variability in their ability to discriminate between participants with and without polyneuropathy. Among the sensory nerves, the right sural nerve exhibited the highest diagnostic accuracy (AUC = 0.766), followed by the right ulnar sensory nerve (AUC = 0.727). Among the motor nerves, the right ulnar motor nerve demonstrated the greatest diagnostic utility (AUC = 0.736). In contrast, several left-sided nerves exhibited lower specificity and reduced overall discriminative ability. Figure 2 shows the ROC curves for individual nerves in the diagnosis of polyneuropathy. Table 1 shows the diagnostic performance of each nerve, including sensitivity, specificity, AUC, and chi-square test.

**Table 1:** Diagnostic performance of individual nerves for polyneuropathy detection.

Nerve	*N*	Sensitivity	Specificity	AUC	*χ*^2^ (*p*)
Sural R	179	72.5%	80.7%	0.766	*χ*^2^ = 50.93***
Sural L	179	84.6%	27.3%	0.559	*χ*^2^ = 3.78*
Ulnar S-R	179	68.1%	77.3%	0.727	*χ*^2^ = 37.15***
Ulnar S-L	179	74.7%	70.5%	0.726	*χ*^2^ = 36.62***
Median S-R	179	53.8%	83.0%	0.684	*χ*^2^ = 26.38***
Median S-L	179	76.9%	39.8%	0.583	*χ*^2^ = 5.80*
Tibial R	182	56.0%	83.5%	0.698	*χ*^2^ = 30.81***
Tibial L	182	64.8%	62.6%	0.637	*χ*^2^ = 13.74***
Ulnar M-R	182	81.3%	65.9%	0.736	*χ*^2^ = 41.62***
Ulnar M-L	182	61.5%	60.4%	0.610	*χ*^2^ = 8.79**
Peroneal R	182	56.0%	75.8%	0.659	*χ*^2^ = 19.24***
Peroneal L	182	40.7%	82.4%	0.615	*χ*^2^ = 11.74***
Median M-R	182	56.0%	67.0%	0.615	*χ*^2^ = 9.81**

S-R/L, sensory right/left; M-R/L, motor right/left. Sensitivity and specificity calculated at the binary classification threshold (nerve abnormal if any measured parameter exceeds reference limits). AUC computed from binary predictor using imputation dataset. Data from 179 (sensory) and 182 (motor) patients. *** *p* < 0.001; ** *p* < 0.01; * *p* < 0.05.

**Figure 3:**
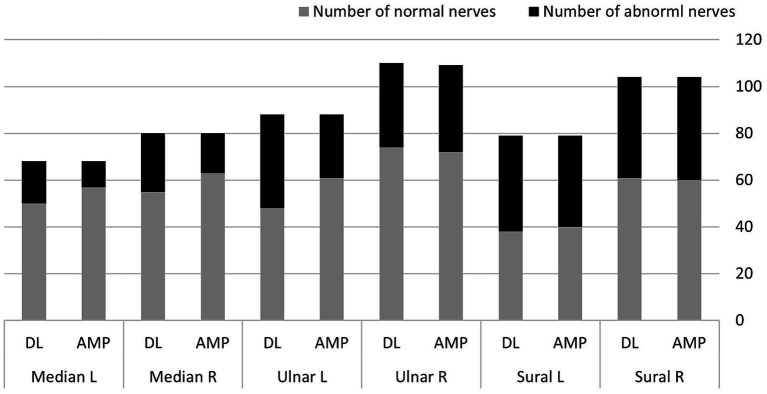
Frequency of Sensory Nerve Abnormalities by Distal Latency and Amplitude. Figure 3 shows the frequency of normal and abnormal sensory nerve findings for distal latency and amplitude in the median, ulnar, and sural nerves on both sides. DL, Distal latency; AMP, Amplitude; R, Right; L, Left.

Figure 3 illustrates the distribution of normal and abnormal sensory nerve findings with a stacked bar chart for distal latency and amplitude measures of the median, ulnar, and sural nerves on the left and right sides. Overall, abnormal findings are more frequently observed in the ulnar and sural nerves compared with the median nerve, with higher counts noted for both measures, particularly on the right side. Distal latency measures tend to show a greater number of abnormalities than amplitude measures across all nerves.

**Figure 4:**
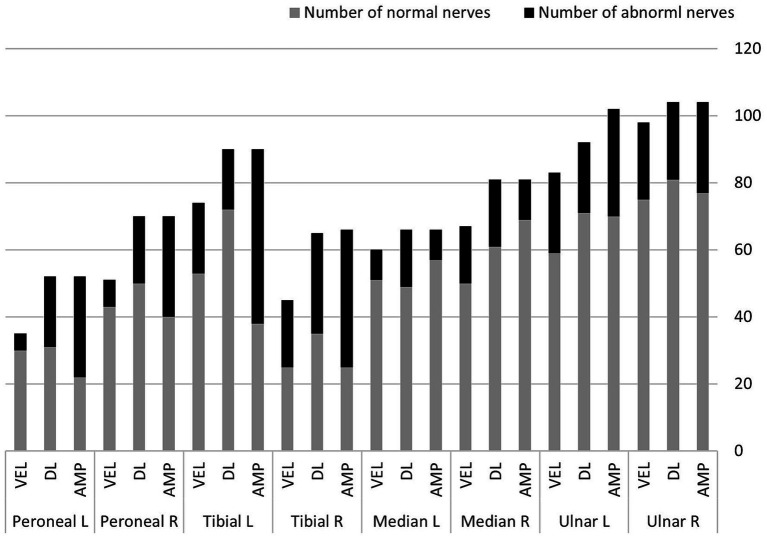
Motor nerves are distributed to normal and abnormal conduction parameters. Figure 4 shows the distribution of normal and abnormal motor nerve conduction findings for conduction velocity, distal latency, and amplitude in the peroneal, tibial, median, and ulnar nerves on both sides. DL, Distal latency; AMP, Amplitude; R, Right; L, Left.

Figure 4 illustrates the distribution of normal and abnormal motor nerve conduction parameters across the peroneal, tibial, median, and ulnar nerves on the left and right sides. The stacked bars show the number of normal and abnormal findings for conduction velocity (VEL), distal latency (DL), and amplitude (AMP). Overall, abnormal conduction findings are more frequently observed in the tibial and ulnar motor nerves, particularly for distal latency and amplitude measures, compared with the peroneal and median nerves. This figure highlights that abnormalities in motor nerve conduction parameters are more prevalent in the tibial and ulnar nerves and supports their greater utility in distinguishing patients with PNP from controls.

Chi-squared (*χ*^2^) test results are presented for sensory nerves in Table 2 and motor nerves in Table 3. The tables include binary indicators of whether each nerve was tested, as well as NCS measures (latency, amplitude, and conduction velocity). Statistically significant results (*p* < 0.05) are shown in bold.

**Table 2:** *χ*^2^ results comparing PNP patients and controls across sensory nerves.

Nerve (sensory)	DL *χ*^2^ (*p*)	AMP *χ*^2^ (*p*)	Binary *χ*^2^ (*p*)
Sural R	**51.5 (<0.001)**	**38.7 (<0.001)**	**21.8 (<0.001)**
Sural L	**38.7 (<0.001)**	0.8 (0.38)	**22.7 (<0.001)**
Ulnar R	**17.8 (<0.001)**	**26.9 (<0.001)**	**36.8 (<0.001)**
Ulnar L	**28.8 (<0.001)**	**21.7 (<0.001)**	**9.7 (0.002)**
Median R	**14.8 (<0.001)**	**15.9 (<0.001)**	**5.7 (0.017)**
Median L	**22.8 (<0.001)**	0.03 (0.85)	0.37 (0.54)

DL, distal latency; AMP, Amplitude; R, right; L, left. Bold values indicate statistically significant differences between PNP patients and controls (*p* < 0.05).

**Table 3:** *χ*^2^ results comparing PNP patients and controls across motor nerves.

Nerve (motor)	DL *χ*^2^ (*p*)	AMP *χ*^2^ (*p*)	VEL *χ*^2^ (*p*)	Binary *χ*^2^ (*p*)
Ulnar R	**96.5 (0.034)**	**98.8 (0.024)**	**103.17 (0.035)**	**41.6 (<0.001)**
Ulnar L	101.1 (0.112)	105.6 (0.064)	114.5 (0.074)	**8.8 (0.003)**
Median R	114.9 (0.91)	108.1 (0.188)	115.9 (0.131)	**9.8 (0.002)**
Median L	130.4 (0.101)	119.4 (0.277)	126.5 (0.258)	0.59 (0.442)
Tibial R	**140.9 (0.029)**	**147.4 (0.012)**	147.2 (0.173)	**30.8 (<0.001)**
Tibial L	104.1 (0.102)	**111.9 (0.037)**	125.2 (0.067)	**13.7 (<0.001)**
Peroneal R	123.4 (0.133)	128.5 (0.077)	133.2 (0.314)	**19.2 (<0.001)**
Peroneal L	144.9 (0.107)	148.1 (0.077)	142.7 (0.466)	**11.7 (<0.001)**

DL, distal latency; AMP, Amplitude; VEL, conduction velocity; R, right; L, left. Bold values indicate statistically significant differences between PNP patients and controls (*p* < 0.05).

Table 2 demonstrates significant group differences across most sensory nerve parameters. The ulnar sensory nerves showed significant bilateral differences across all parameters, with strong associations for distal latency and amplitude. For the median sensory nerves, all parameters were significant unilaterally, whereas only distal latency was significant on the contralateral side. In the sural nerves, both sides demonstrated highly significant differences in distal latency and binary outcomes, while amplitude was significant on one side.

The superficial peroneal and radial (sensory and motor) nerves were included only as binary parameters (tested vs. not tested) and were therefore excluded from the tables to improve readability.

Among the 15 participants with recorded abnormal right superficial peroneal findings, 14 had polyneuropathy, yielding a significant association on chi-square analysis (*χ*^2^ = 12.27, *p* < 0.001). No significant associations were observed for the right (*χ*^2^ = 0.871, *p* = 0.351) and left radial sensory nerve (*χ*^2^ = 4.25, *p* = 0.390), as well as the left superficial peroneal nerve (*χ*^2^ = 3.07, *p* = 0.080). For the radial motor nerves, chi-squared analysis showed no significant associations for either the right side (*χ*^2^ = 0.87, *p* = 0.35) or the left side (*χ*^2^ = 0.15, *p* = 0.70).

Table 3 shows statistically significant binary associations for the ulnar, tibial, and peroneal motor nerves bilaterally. Among continuous measures, the right ulnar motor nerve demonstrated significant differences across all parameters (distal latency, amplitude, and conduction velocity), while the tibial nerves showed significant differences mainly in amplitude on both sides and in distal latency on the right. In contrast, the median and peroneal motor nerves did not exhibit significant differences in continuous parameters despite significant binary associations.

For the sensory nerves, the logistic regression model was statistically significant [*χ*^2^(16) = 274.64, *p* < 0.001], explaining 60.7% of the variance in PNP (Nagelkerke *R*^2^) and correctly classifying 83.7% of cases. Binary sensory nerve abnormalities of the sural and ulnar nerves were strong predictors, showing significant bilateral associations with large odds ratios as shown in Table 4. In contrast, median sensory nerve abnormalities were not significantly associated with PNP. Among continuous measures, only the right sural distal latency and right ulnar amplitude reached statistical significance, while most sensory amplitudes and distal latencies did not. Overall, these findings indicate that binary sensory nerve abnormalities, particularly in the sural and ulnar nerves, are more informative than individual continuous sensory conduction measures for distinguishing PNP patients from controls.

**Table 4:** Logistic regression estimates of the sensory nerves.

Nerve	Side	B	S. E.	*P*	Exp (B)
**Sural**	**Right**	**2.304**	**0.312**	**<0.001**	**10.013**
	**Left**	**2.060**	**0.334**	**<0.001**	**7.842**
Sural AMP	Right	0.352	0.490	0.481	1.423
	Left	−0.172	0.429	0.693	0.842
Sural DL	Right	**1.2**	**0.590**	**0.043**	**3.279**
	Left	0.728	0.717	0.310	2.071
**Ulnar**	**Right**	**2.238**	**0.308**	**<0.001**	**9.370**
	**Left**	**1.589**	**0.322**	**<0.001**	**4.899**
Ulnar AMP	**Right**	**1.185**	**0.505**	**0.019**	**3.272**
	Left	0.085	0.576	0.886	1.089
Ulnar DL	Right	0.384	0.377	0.310	1.468
	Left	0.716	0.919	0.450	2.047
Median	Right	0.201	0.316	0.061	1.223
	Left	0.124	0.588	0.837	1.132
Median AMP	Right	1.218	0.651	0.061	3.379
	Left	−3.21	0.456	0.481	0.725
Median DL	Right	0.142	0.454	0.760	1.152
	Left	0.193	0.485	0.699	1.213

DL, distal latency; AMP, Amplitude. Bold values indicate statistically significant differences between PNP patients and controls (*p* < 0.05).

For the motor nerves, the model was statistically significant *χ*^2^(11) = 38.32, *p* < 0.001, explaining 10.8% of the variance in PNP, and correctly classifying 64.4% of the cases (see Table 5). However, none of the variables were found to be statistically significant in differentiating the groups.

**Table 5:** Logistic regression estimates of the motor nerves.

Nerve	Side	B	S.E.	P	Exp (B)
Median VEL	Right	0.237	0.362	0.525	1.267
	Left	0.165	0.362	0.657	1.179
Median AMP	Left	0.048	0.378	0.900	1.050
Ulnar AMP	Right	0.104	0.284	0.715	1.110
Ulnar DL	Right	0.344	0.304	0.268	1.411
Ulnar VEL	Right	0.134	0.346	0.703	1.144
Tibial AMP	Right	0.222	0.281	0.436	1.249
	Left	0.345	0.388	0.389	1.413
Tibial VEL	Right	0.036	0.324	0.912	1.037
	Left	0.244	0.281	0.390	1.276
Peroneal AMP	Left	0.238	0.284	0.409	1.269

DL, distal latency; AMP, Amplitude; VEL, conduction velocity; R, right; L, left.

**Figure 5:**
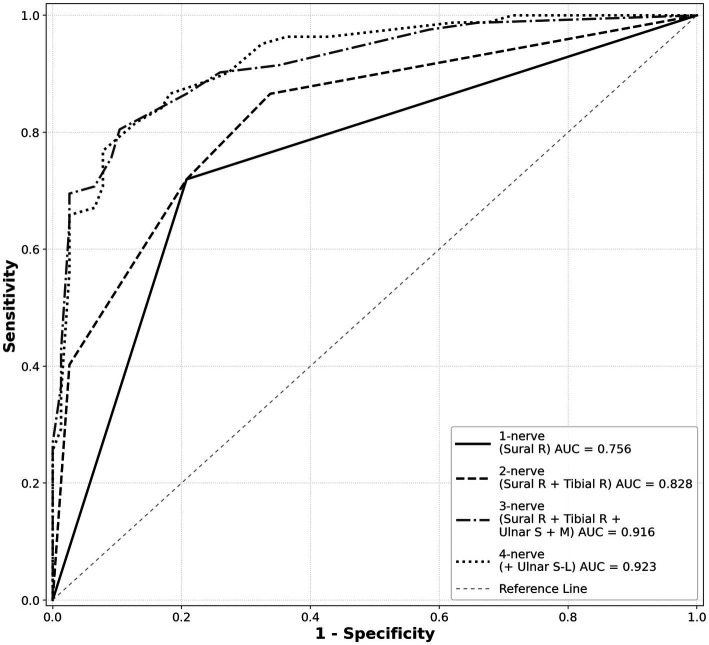
ROC Comparison of Progressively Expanded Nerve-Testing Protocols. Receiver operating characteristic (ROC) curves compare the diagnostic performance of progressively expanded nerve-testing protocols. The one-nerve protocol included the right sural sensory nerve; the two-nerve protocol added the right tibial motor nerve; and the three-nerve protocol added right ulnar sensory and motor studies. The expanded protocol additionally included the left ulnar sensory nerve. Curves were generated from logistic regression predicted probabilities. Stars indicate the optimal operating points. R, right; L, left; S, sensory; M, motor.

ROC analyses of progressively expanded diagnostic protocols demonstrated improvements in diagnostic performance as additional nerves were incorporated, as shown in Figure 5. The single-nerve protocol based on the right sural nerve demonstrated moderate diagnostic accuracy (sensitivity = 72.0%, specificity = 79.2%, AUC = 0.756). The addition of the right tibial nerve increased sensitivity (86.6%) and overall discrimination (AUC = 0.828), although specificity decreased (66.2%). The proposed protocol, consisting of the right sural nerve, right tibial nerve, right ulnar sensory nerve, and right ulnar motor nerve, demonstrated excellent diagnostic performance, with a sensitivity of 80.5%, specificity of 89.6%, and an AUC of 0.916. Although the addition of the left ulnar sensory nerve resulted in a slight increase in specificity (92.2%) and AUC (0.923), sensitivity decreased to 76.8%, suggesting only marginal diagnostic benefit beyond the proposed protocol. Table 6 shows the diagnostic performance of the nerve testing protocol.

**Table 6:** Diagnostic performance by nerve testing protocol.

Protocol	n	Sensitivity	Specificity	AUC
1-nerve (sural R)	162	0.720	0.792	0.756
2-nerve (sural R + Tibial R)	162	0.866	0.662	0.828
3-nerve (sural R + Tibial R + Ulnar S-R + Ulnar M-R)	162	0.805	0.896	0.916
4-nerve (3-nerve + Ulnar S-L)	162	0.768	0.922	0.923

Area Under the ROC Curve (AUC) calculated using logistic regression predicted probabilities. Sensitivity and specificity values represent optimal operating points determined by Youden Index maximization. Recommended 3-nerve protocol includes: Sural sensory (right), Tibial motor (right), and Ulnar nerve (sensory and motor, bilateral assessment). *n* = 162 patients (82 with polyneuropathy, 77 control subjects).

## Discussion

Our study aimed to identify the minimum number of nerves required to achieve high sensitivity and overall diagnostic accuracy while retaining adequate specificity. Such a protocol may reduce false-negative diagnoses, shorten examination time, and optimize resource use. This objective is supported by variability in guideline adherence and test selection (7), and by evidence that appropriate nerve selection is essential for diagnostic accuracy and efficiency (25). Nevertheless, few studies have examined the minimum number of nerves required to diagnose polyneuropathy (10).

The study hypothesized that examining two motor nerves and two sensory nerves would be sufficient to achieve a level of certainty in diagnosis. The hypothesis was supported. The three-nerve protocol, comprising sural sensory, tibial motor, and ulnar sensory and motor studies, achieved high diagnostic performance. Sequential ROC analysis showed that the sural nerve alone provided moderate discrimination. Adding the tibial nerve increased sensitivity but reduced specificity, whereas adding ulnar sensory and motor studies produced a more favorable balance (AUC = 0.916; sensitivity = 80.5%; specificity = 89.6%). Adding contralateral ulnar sensory testing produced only a marginal increase in AUC to 0.923 and was accompanied by reduced sensitivity. These findings are consistent with evidence recommending initial screening with sural sensory and tibial motor studies and requiring abnormalities in at least two nerves to support the diagnosis of polyneuropathy (12). This supports its use as a focused approach to nerve selection that may simplify polyneuropathy assessment while maintaining diagnostic performance.

If doubts arise, an additional nerve can be introduced to eliminate another differential diagnosis or reinforce the test results. For this reason, and because the clinician conducting the electrodiagnostic test evaluates all parameters of the nerve to distinguish between demyelinating and axonal disorders, the guidelines we recommend focus on which nerves to examine rather than specific parameters.

The findings indicate that unilateral testing of the sural and ulnar sensory nerves provided substantial diagnostic information, with right-sided measures showing stronger diagnostic associations than corresponding left-sided measures. These nerves showed high rates of abnormal findings and strong group discrimination on chi-square analysis. They also had independent predictive value in logistic regression (Table 4). However, the side-specific analyses were intended to evaluate whether unilateral testing may provide adequate diagnostic confidence and how much additional value is gained by testing the contralateral side, rather than to examine biological asymmetry. Although the right side showed higher diagnostic performance in this cohort, this should not be interpreted as evidence of true right–left asymmetry or as a recommendation to preferentially test the right side. Instead, the findings suggest that bilateral testing may increase diagnostic confidence, but the added value of the second side may be relatively lower.

For the sural sensory nerve, chi-square analysis showed strong bilateral associations for distal latency (right *χ*^2^ = 51.5, *p* < 0.001, left *χ*^2^ = 38.7, *p* < 0.001) and binary outcomes (right *χ*^2^ = 21.8, *p* < 0.001, left *χ*^2^ = 22.7, *p* < 0.001), with amplitude significant on the right side only (*χ*^2^ = 38.7, *p* < 0.001). Correspondingly, the sural nerve demonstrated the largest effect sizes in logistic regression (right OR = 10.0, *p* < 0.001, left OR = 7.8, *p* < 0.001). Similarly, the ulnar sensory nerve exhibited significant bilateral chi-square associations across all parameters, including distal latency (right *χ*^2^ = 17.8, *p* < 0.001, left *χ*^2^ = 28.8, *p* < 0.001), amplitude (right *χ*^2^ = 26.9, *p* < 0.001, left *χ*^2^ = 21.7, *p* < 0.001), and binary status (right *χ*^2^ = 36.8, *p* < 0.001, left *χ*^2^ = 9.7, *p* = 0.002), along with large odds ratios in logistic regression (right OR = 9.4, *p* < 0.001, left OR = 4.9, *p* < 0.001). In contrast, the median sensory nerve showed less consistent findings, with chi-square significance largely limited to one side and no independent predictive value in logistic regression. The results indicate that both sural and ulnar sensory nerves are important for the diagnosis of polyneuropathy, as abnormalities on either side were independently associated with PNP. The presence of normal findings in the contralateral sural and ulnar sensory nerves may therefore be associated with a lower likelihood of PNP and should prompt consideration of alternative diagnoses, particularly in the absence of other supportive electrophysiologic abnormalities.

The median sensory nerve was less informative than the sural and ulnar sensory nerves for the diagnosis of polyneuropathy. However, distal latency showed significant associations, most notably on the right side. Median nerve amplitudes were not consistently significant, and the median sensory nerve did not demonstrate independent predictive value in logistic regression. The superficial peroneal sensory nerve showed a significant association only as a binary variable on the right side, while its distal latency and amplitude measures were excluded from analysis because of a high frequency of missing values. This association was driven by recorded abnormal findings, as 14 of the 15 participants with abnormal right superficial peroneal findings had polyneuropathy. Because the remaining cases largely represented untested or missing values rather than confirmed normal studies, this result reflects a higher occurrence of abnormal superficial peroneal findings among participants with polyneuropathy rather than diagnostic sensitivity or specificity. Overall, this nerve contributed less to direct diagnostic discrimination in the present analysis. However, its assessment may still be valuable when considering alternative, asymmetric, or focal neuropathic processes. This supports its role in the evaluation of differential diagnoses rather than primary PNP identification. Motor nerve abnormalities, most frequently observed in the tibial and ulnar nerves, showed significant binary associations on chi-square analysis (tibial: right *χ*^2^ = 30.8, *p* < 0.001; left *χ*^2^ = 13.7, *p* < 0.001; ulnar: right *χ*^2^ = 41.6, *p* < 0.001; left *χ*^2^ = 8.8, *p* = 0.003). Among continuous motor parameters, significant chi-square differences were observed for distal latency and amplitude in selected nerves. The right ulnar motor nerve demonstrated significant differences across distal latency (*χ*^2^ = 96.5, *p* = 0.034), amplitude (*χ*^2^ = 98.8, *p* = 0.024), and conduction velocity (*χ*^2^ = 103.2, *p* = 0.035), while the tibial motor nerve showed significant amplitude differences bilaterally (right *χ*^2^ = 147.4, *p* = 0.012; left *χ*^2^ = 111.9, *p* = 0.037) and a significant distal latency difference on the right side (*χ*^2^ = 140.9, *p* = 0.029). Despite these significant associations, no motor nerve variables remained independent predictors in logistic regression, and the motor model explained substantially less variance than the sensory model (Nagelkerke *R*^2^ = 10.8% vs. 60.7%).

Based on consistent evidence from frequency analyses, chi-square testing, multivariable logistic regression, and sequential ROC analysis, a structured and stepwise NCS approach is proposed for the evaluation of suspected polyneuropathy. Sensory nerve abnormalities involving the sural and ulnar nerves demonstrated the strongest and most consistent diagnostic associations, while motor nerve abnormalities of the tibial and ulnar nerves provided supportive diagnostic information.

The side-specific analyses support a stepwise approach. Testing the selected nerves unilaterally provides an informative initial assessment, while subsequent contralateral testing helps demonstrate bilateral involvement and increases confidence that the findings represent a generalized polyneuropathy.

**Figure 6:**
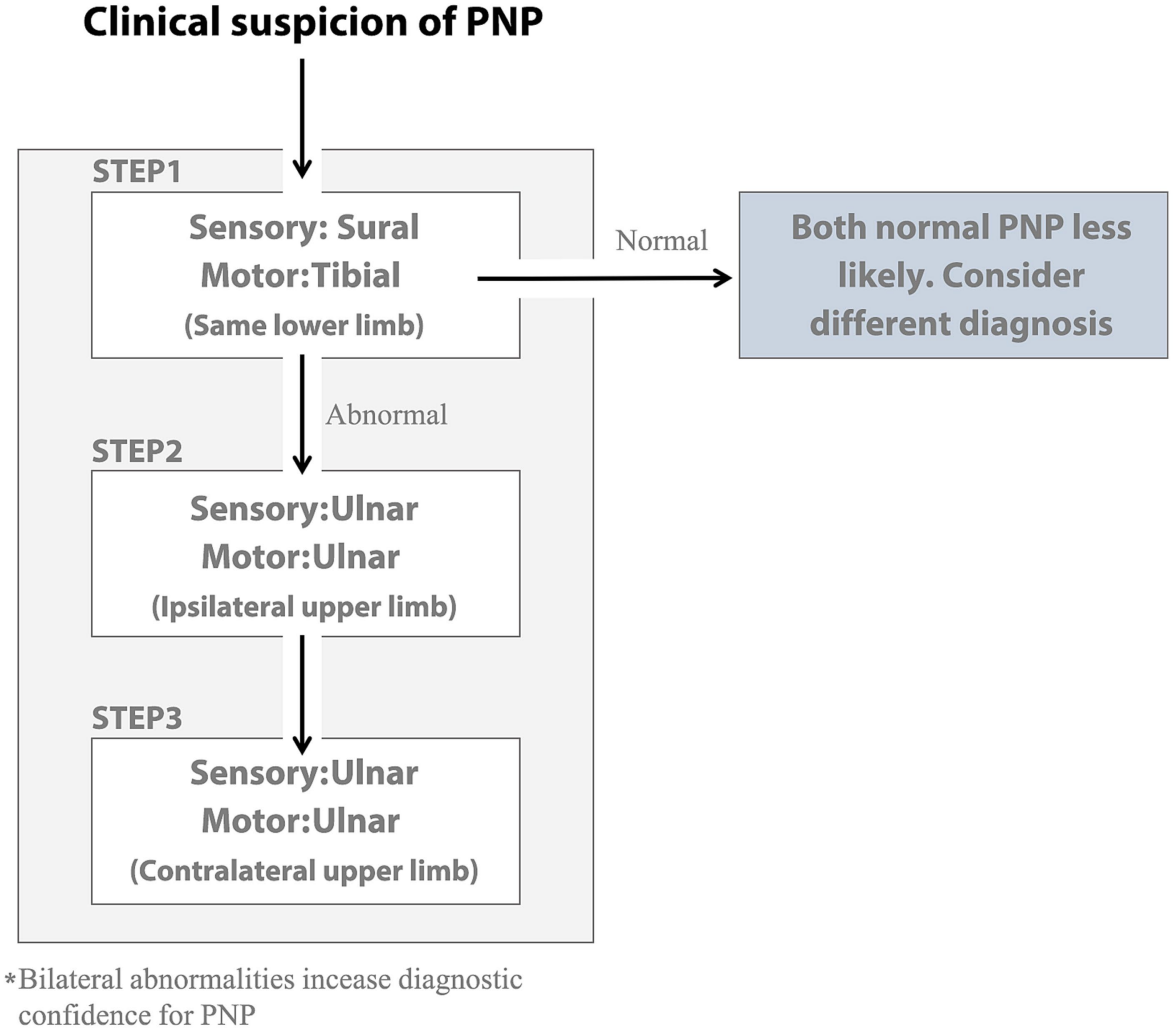
Three suggested steps for PNP diagnosis. Figure 6 illustrates a stepwise nerve conduction study approach for suspected polyneuropathy. Initial evaluation should include sural sensory and tibial motor studies in the same lower limb. If findings are abnormal or equivocal, ulnar sensory and ulnar motor studies should be added in the ipsilateral upper limb, followed by the contralateral upper limb to assess bilateral involvement and increase diagnostic confidence.

The following recommendations are illustrated in Figure 6:

1. Sural sensory and tibial motor nerve conduction studies should be performed on the same lower limb as the initial evaluation. Abnormal or absent findings in either nerve support the diagnosis of polyneuropathy and justify further upper limb assessment. When both sural sensory and tibial motor studies are normal, polyneuropathy is less likely, and alternative diagnoses should be considered in the appropriate clinical context.
2. If lower limb findings are abnormal or equivocal, ulnar sensory and ulnar motor nerve conduction studies should be performed in the upper limb on the same side. Abnormalities in these studies further strengthen the likelihood of polyneuropathy. If the ulnar sensory and motor studies are normal despite abnormal or equivocal lower-limb findings, contralateral lower-limb studies or additional clinically selected nerves should be examined to confirm a generalized process and exclude focal abnormalities. Normal ulnar findings do not exclude an early length-dependent polyneuropathy.
3. Ulnar sensory and ulnar motor nerve conduction studies should then be performed on the contralateral upper limb to assess for bilateral involvement, which increases diagnostic confidence and supports a generalized neuropathic process.

Our model aligns with recent evidence suggesting that multiple abnormal findings across at least two to four nerves are necessary to confidently diagnose polyneuropathy and assess severity (2, 26). In line with this, a recent scoring system was developed by Stepien and Pastuszak (27) based on electroneurography, reinforces the clinical value of testing a minimal yet diverse set of nerves, specifically highlighting the correlation between the number of abnormal nerves and neuropathy severity. We recommend starting with the sural nerve, consistent with prior studies on PNP electrodiagnosis (10, 12, 28, 29). We also believe the tibial F-wave alone lacks specificity and may overlook conditions such as radiculopathy or plexopathy, as suggested in previous research (4). While the minimal criteria for DSP proposed by AANEM focus on the peroneal motor and sural sensory nerves, the broader AANEM guidelines for electrodiagnostic evaluation recommend testing a more comprehensive set of nerves, typically 4 motor and 4 sensory nerves across both legs and one arm. Importantly, these broader recommendations are based largely on expert consensus rather than high-level evidence (30). An older study included data from six different centers in six different European countries where electrodiagnostics is performed and included about 1,400 patients, of whom about 350 had polyneuropathy. The study’s recommendations indicate that a minimum of two motor nerves and two sensory nerves are sufficient for diagnosis. The study did not discuss which nerves were preferred, but it was a milestone in the diagnosis of polyneuropathy (31). Another study aimed at simplifying the NCS findings for patients with diabetes, as a criterion for diagnosing sensory-motor polyneuropathy, recommended testing three primary nerves: the sural, tibial, and peroneal nerves. The study did not include the examination of the upper limbs. The results indicated that the tibial F-wave, the conduction velocity of the peroneal nerve, and the sum of the conduction velocities can predict sensory-motor diabetic neuropathy after 4 years with a sensitivity of 80% and specificity of 70%^28.^

To answer the contradiction between some of the current guidelines, which include the NCS of the motor peroneal nerve (2), and others that suggest testing the tibial nerve (10) we found higher confidence in examining the tibial nerve as mentioned in Table 3. Tankisi et al. demonstrated high sensitivity in assessing the ulnar and median nerves; however, they advised that these findings should not be used for the diagnosis of polyneuropathy per se, but rather for distinguishing between axonal and demyelinating subtypes (12).

Previous research addresses the need to clarify whether assessment of a single limb is sufficient for each nerve, as this issue has no clear answer (9), our study provides an answer to this issue in the form of the suggested model. According to our approach, examining both upper and lower limbs is important for a high level of certainty in the diagnosis. Conducting NCS solely on the lower limbs may lead to false negative diagnoses due to other prevalent conditions, such as radiculopathy, which is also observed (4). Moreover, patients with PNP might have comorbidities, they could suffer from additional abnormalities that are treated differently, and missing those might result in health deterioration and disability that otherwise might be prevented. We adopted the recommendation to include the ulnar nerve, as its considerable length in the upper limb makes it likely to be affected in length-dependent polyneuropathy (32). Additionally, Galiero et al. (11) demonstrated that incorporating the whole plantar nerve may improve early detection of distal neuropathies, suggesting that careful nerve selection can boost sensitivity in subclinical cases. Our proposed triad thus adheres to emerging consensus recommendations while offering practical feasibility for routine clinical use.

## Limitations

Research limitations include the lack of sufficient representation of the number of nerves that could potentially increase the accuracy of a diagnosis with polyneuropathy, such as the dorsal sural nerve, medial, or lateral plantar nerves (12).

However, the study goal was to determine the minimum number of nerves needed to diagnose polyneuropathy, so adding nerves would make the model more complex and less efficient. Including additional nerves may increase diagnostic sensitivity in selected cases, but would also increase protocol complexity and reduce efficiency. Nerves that were performed selectively rather than routinely were analyzed as tested/not-tested variables when appropriate, but were not included in the proposed protocol unless they demonstrated consistent diagnostic contribution in the matched cohort. Accordingly, the final protocol focuses on nerves with more robust and reproducible associations with PNP.

Another limitation is the use of the ±2 SD rule to define abnormal parameters, which is widely accepted as the conventional statistical standard. However, rigid application of this cutoff inevitably introduces a small but real risk of false positives and negatives, particularly when multiple tests are performed. Thus, interpretation should always be supported by clinical correlation and by considering consistency across tests.

In addition, although several complementary analyses (chi-square tests, multivariable logistic regression, and ROC analyses) supported the diagnostic utility of the proposed protocol for accurately diagnosing polyneuropathy using a minimal number of nerve studies, the study was not designed as a non-inferiority trial. The sequential protocol analysis showed only a small increase in AUC when a fourth nerve was added to the three-nerve protocol (0.923 vs. 0.916). Formal non-inferiority, therefore, requires prospective confirmation. Nevertheless, our real-world data provide consistent evidence supporting the efficiency of the proposed three-nerve approach.

Additionally, electrophysiological testing is restricted to large-fiber neuropathies, and small-fiber neuropathies cannot be detected with this methodology. As such, cases of isolated small-fiber neuropathy may have been overlooked in our analysis. Further research is required in order to prospectively examine the accuracy of the model. External validation in independent and diverse patient cohorts is required to confirm the diagnostic performance and generalizability of the proposed protocol before its broader clinical implementation. Additionally, using data to predict polyneuropathy as a diagnosis through statistical methods, such as machine learning techniques, can provide further insights.

## Conclusion

The effectiveness and reliability of a nerve conduction test in diagnosing polyneuropathy significantly depend on the number of nerves examined. This study suggests that testing three specific nerves: the sural nerve, the tibial nerve, and the ulnar nerve (sensory and motor), is sufficient for diagnosing polyneuropathy.

## Data Availability

The individual-level clinical lectrodiagnostic dataset is not publicly available due to patient privacy and ethical restrictions. Deidentified data may be made available from the corresponding author upon reasonable request and subject to institutional approval and applicable ethical and regulatory requirements.
