# Supplementary material for: Efficient electrodiagnosis of polyneuropathy—a targeted three-nerve conduction study protocol
Source: Front Neurol. 2026 Jul 28;17:1862881. doi: 10.3389/fneur.2026.1862881 (PMC13457068; doi:10.3389/fneur.2026.1862881)
Supplement: Supplementary file 1 [file Table_1.DOCX]

**Supplementary Table S1**

**Nerve conduction study protocol and published reference limits used for classification**

# A. General protocol

| **Protocol item** | **Study specification** |
| --- | --- |
| **EMG/NCS system** | Nicolet VikingQuest two-channel EMG machine \| Software: [Viking Software A/S, Odense, Denmark] |
| **Motor filter settings** | 3 Hz to 10 kHz |
| **Sensory filter settings** | 20 Hz to 2 kHz |
| **Motor sweep and sensitivity** | 2-3 ms/div; 5 mV/div |
| **Sensory sweep and sensitivity** | 1-2 ms/div; 20 µV/div |
| **Temperature requirements** | Hand ≥ 32°C; foot ≥ 30°C (skin surface at recording site) |
| **Stimulation** | Supramaximal (10%–30% above current required for maximal CMAP/SNAP); surface stimulating electrodes; 0.1-0.2 ms pulse duration; 0–100 mA range |
| **Amplitude measurement** | CMAP: baseline–to–negative peak (mV); SNAP: baseline-to-negative peak (µV) |
| **Latency measurement** | Onset latency for DML and conduction velocity; peak latency for SNAP |
| **Sides examined** | Right side (standard); bilateral, where clinically indicated or for comparison |
| **Reference-value basis** | Published normative data; not derived from the present study cohort |

# B. Sensory nerve conduction studies

| **Nerve** | **Protocol: side, recording/stimulation sites, distance, technique** | **Parameter** | **Published reference limit** | **Abnormal classification** | **source** |
| --- | --- | --- | --- | --- | --- |
| **Median sensory** | Side: right (Lt if indicated); record: ring electrode, proximal phalanx digit II or III; stimulation: wrist, 14 cm proximal to E1, between FCR and palmaris longus tendons; antidromic | SNAP amplitude (microV) | >= 15 µV | < 15 µV | Ref. 1 |
|  |  | Peak latency (ms) | <= 3.7 ms (peak) | > 3.7 ms | Ref. 2 |
|  |  |  |  |  |  |
| **Ulnar sensory** | Side: right (Lt if indicated); record: ring electrode, proximal phalanx digit V; stimulation: wrist, 14 cm proximal to E1, near FCU tendon; antidromic | SNAP amplitude (microV) | >= 10 µV | < 10 µV | Ref. 3 |
|  |  | Peak latency (ms) | <= 3.2 ms (peak) | > 3.2 ms | Ref. 4 |
|  |  |  |  |  |  |
| **Radial sensory** | Side: right (Lt if indicated); record: anatomical snuffbox (between EPL and EPB tendons); stimulation: lateral forearm over radius, 12–14 cm proximal to E1; antidromic | SNAP amplitude (microV) | >= 15 µV | < 15 µV | Ref. 5 |
|  |  | Peak latency (ms) | <= 3.7 ms (peak) | > 3.7 ms | Ref. 6 |
|  |  |  |  |  |  |
| **Sural sensory** | right (Lt if indicated); record: posterior to lateral malleolus; stimulation: posterolateral calf, 14 cm proximal to E1, lateral to Achilles tendon; antidromic | SNAP amplitude (microV) | >= 6 µV | < 6 µV | Ref. 7 |
|  |  | Peak latency (ms) | <= 4.2 ms (peak, 14 cm) | > 4.2 ms | Ref. 8f |
|  |  |  |  |  |  |
| **Superficial peroneal sensory** | Side: right (Lt if indicated); record: 1–2 cm medial to lateral malleolus; stimulate: anterior to fibula edge, 14 cm proximal to E1; antidromic | SNAP amplitude (microV) | >= 6 µV | < 6 µV | Ref. 9f |
|  |  | Peak latency (ms) | <= 4.2 ms (peak, 14 cm) | > 4.2 ms | Ref. 10 |

# C. Motor nerve conduction studies

| **Nerve** | **Protocol: side, recording/stimulation sites, distance, technique** | **Parameter** | **Published reference limit** | **Abnormal classification** | **Adjustment and source** |
| --- | --- | --- | --- | --- | --- |
| **Median motor** | Side: right (Lt if indicated); record: APB belly (E1), thumb proximal phalanx (E2); stimulation: wrist 8 cm proximal to E1 between FCR/palmaris longus, then elbow (antecubital fossa); belly-tendon montage | CMAP amplitude (mV) | >= 4.0 mV | < 4.0 mV | Ref.11 |
|  |  | Distal motor latency (ms) | <= 4.4 ms | > 4.4 ms | Ref. 12 |
|  |  | Conduction velocity, Forearm (m/s) | >= 49 m/s | < 49 m/s | Ref. 13 |
|  |  |  |  |  |  |
| **Ulnar motor** | Side: right (Lt if indicated); record: ADM belly (E1), digit V proximal phalanx (E2); stimulation: wrist 8 cm proximal to E1 near FCU, below elbow (3–4 cm distal to medial epicondyle), above elbow ( 10 cm in total, proximal); 90° elbow flexion; belly-tendon montage | CMAP amplitude (mV) | >= 5.0 mV | < 5.0 mV | Ref. 14s |
|  |  | Distal motor latency (ms) | <= 3.6 ms | > 3.6 ms | Ref. 15 |
|  |  | Conduction velocity, Forearm/across elbow (m/s) | >= 49 m/s | < 49 m/s | Ref. 16 |
|  |  |  |  |  |  |
| **Radial motor** | Side: right (Lt if indicated); record: extensor indicis proprius (EIP) belly (E1), MCP joint of index finger (E2); stimulate: forearm (posterior interosseous nerve, 8 cm proximal to E1, lateral to radius); belly-tendon montage | CMAP amplitude (mV) | >= 1.8 mV | < 1.8 mV | Ref. 17 |
|  |  | Distal motor latency (ms) | <= 3.1 ms (forearm to EIP, 8 cm) | > 3.1 ms | Ref. 18 |
|  |  | Conduction velocity, Forearm/spiral groove (m/s) | >= 49 m/s | < 49 m/s | Ref. 19 |
|  |  |  |  |  |  |
| **Peroneal/fibular motor** | Side: right (Lt if indicated); record: EDB belly (E1), 5th MTP joint (E2); stimulate: ankle 8 cm proximal to E1 lateral to tibialis anterior tendon, below fibular head, popliteal fossa; belly-tendon montage | CMAP amplitude (mV) | >= 2.0 mV | < 2.0 mV | Ref. 20 |
|  |  | Distal motor latency (ms) | <= 6.2 ms (ankle, 8 cm) | > 6.2 ms | Ref. 21 |
|  |  | Conduction velocity, Leg/across fibular head (m/s) | >= 40 m/s | < 40 m/s | Ref. 22 |
|  |  |  |  |  |  |
| **Tibial motor** | Side: right (Lt if indicated); record: abductor hallucis belly 1 cm below/behind navicular tubercle (E1), 1st MTP joint (E2); stimulate: ankle posterior to medial malleolus, popliteal fossa; belly-tendon montage | CMAP amplitude (mV) | >= 4.0 mV | < 4.0 mV | Ref. 23 |
|  |  | Distal motor latency (ms) | <= 6.0 ms (ankle, 8 cm) | > 6.0 ms | Ref. 24 |
|  |  | Conduction velocity, Leg (m/s) | >= 40 m/s | < 40 m/s | Ref. 25 |
|  |  |  |  |  |  |

Notes; CMAP, compound muscle action potential; DML, distal motor latency; LLN, lower limit of normal; NCS, nerve conduction study; SNAP, sensory nerve action potential; ULN, upper limit of normal.

**References**

**Source for all DeLisa chapter references below - Lee HJ, DeLisa JA. Manual of Nerve Conduction Study and Surface Anatomy for Needle Electromyography, 4th ed. Philadelphia: Lippincott Williams & Wilkins; 2005.**

1. Johnson EW, Melvin JL. Sensory conduction studies of median and ulnar nerves. Arch Phys Med Rehabil. 1967;48:25–30. [Lee HJ, DeLisa JA. Ch. 3, Ref. 7. + (KUMC unpublished, same chapter) + Buschbacher RM, Prahlow ND (eds). Manual of Nerve Conduction Studies, 2nd ed. New York: Demos Medical Publishing; 2006.

2. Johnson EW, Melvin JL. Sensory conduction studies of median and ulnar nerves. Arch Phys Med Rehabil. 1967;48:25–30. [Lee HJ, DeLisa JA. Ch. 3, Ref. 22

3. Korea University Medical Center (KUMC), unpublished data. [Lee HJ, DeLisa JA. Ch. 3, Ref. 27.

4. Johnson EW, Melvin JL. Sensory conduction studies of median and ulnar nerves. Arch Phys Med Rehabil. 1967;48:25–30. [Lee HJ, DeLisa JA. Ch. 3, Ref. 22

5. Johnson EW, Melvin JL. Sensory conduction studies of median and ulnar nerves. Arch Phys Med Rehabil. 1967;48:25–30. [Lee HJ, DeLisa JA. Ch. 3, Ref. 22.

6. Johnson EW, Melvin JL. Sensory conduction studies of median and ulnar nerves. Arch Phys Med Rehabil. 1967;48:25–30. [Lee HJ, DeLisa JA. Ch. 3, Ref. 22.

7. Korea University Medical Center (KUMC), unpublished data. [Lee HJ, DeLisa JA. Ch. 3, Ref. 27.

8. Schuchmann JA. Sural nerve conduction: a standardized technique. Arch Phys Med Rehabil. 1977;58(4):166–168. [Lee HJ, DeLisa JA. Ch. 4, Ref. 15. + Korea University Medical Center (KUMC), unpublished data, same chapter.

9. Jabre JF. [No full citation provided in Lee HJ, DeLisa JA. Ch. 4, Ref. 5.

10. Izzo KL et al. [No full citation provided in Lee HJ, DeLisa JA. Ch. 4, Ref. 6.

11. Korea University Medical Center (KUMC), unpublished data. [Lee HJ, DeLisa JA. Ch. 3, + KUMC unpublished

12. Buschbacher RM. [No full citation provided in Lee HJ, DeLisa JA. Ch. 3, Ref. 16.

13. Trojaborg W, Sindrup EH. [Lee HJ, DeLisa JA. Ch. 3, Ref. 26.

14. Korea University Medical Center (KUMC), unpublished data. [Lee HJ, DeLisa JA. Ch. 4.

15. Fu R et al. [Lee HJ, DeLisa JA. Ch. 4, Ref. 10.]

16. Korea University Medical Center (KUMC), unpublished data. [Lee HJ, DeLisa JA. Ch. 7, Normal Values 7.

17. Korea University Medical Center (KUMC), unpublished data. [Lee HJ, DeLisa JA. Ch. 7, Normal Values 8.

18. Trojaborg W, Sindrup EH. [Lee HJ, DeLisa JA. Ch. 3, Ref. 26.

19. Jebsen RH. [Lee HJ, DeLisa JA. Ch. 3, Ref. 25; + [Ref. 26].

20. Korea University Medical Center (KUMC), unpublished data. [Lee HJ, DeLisa JA. Ch. 4, Ref. 1.

21. Korea University Medical Center (KUMC), unpublished data. [Lee HJ, DeLisa JA. Ch. 4, Ref. 1.

22. Korea University Medical Center (KUMC), unpublished data. [Lee HJ, DeLisa JA. Ch. 4, Ref. 1.

23. Fu R et al. [Lee HJ, DeLisa JA. Ch. 4, Ref. 10.

24. Fu R et al. [Lee HJ, DeLisa JA. Ch. 4, Ref. 10.

25. Fu R et al. [Lee HJ, DeLisa JA. Ch. 4, Ref. 10.
