# Supplementary material for: Efficient electrodiagnosis of polyneuropathy—a targeted three-nerve conduction study protocol
Source: Front Neurol. 2026 Jul 28;17:1862881. doi: 10.3389/fneur.2026.1862881 (PMC13457068; doi:10.3389/fneur.2026.1862881)
Supplement: Supplementary file 2 [file Table_2.DOCX]

**Appendix**

**Table S1: patients' characteristics before matching**

|  | | | | |
| --- | --- | --- | --- | --- |
|  | **PNP (N=259)** | **Normal (N=196)** | **Total (N=455)** | **p value** |
| **Age** |  |  |  | **< 0.001** |
| Mean (SD) | 62.3 (16.2) | 38.2 (15.5) | 51.9 (19.9) |  |
| Mean (CI) | 62.3 (60.3, 64.3) | 38.2 (36, 40.4) | 51.9 (50.1, 53.7) |  |
| Median (Q1, Q3) | 65.9 (52.0, 73.3) | 36.0 (24.2, 48.4) | 52.9 (35.3, 68.9) |  |
| **Gender** |  |  |  | **< 0.001** |
| Male | 178 (68.7%) | 84 (42.9%) | 262 (57.6%) |  |
| Female | 81 (31.3%) | 112 (57.1%) | 193 (42.4%) |  |

**Table S2: patients' characteristics after matching**

|  | | | | |
| --- | --- | --- | --- | --- |
|  | **PNP (N=91)** | **Normal (N=91)** | **Total (N=182)** | **p value** |
| **Age** |  |  |  | P>0.05 |
| Mean (SD) | 48.9 (17.5) | 46.5 (16.1) | 47.7 (16.8) |  |
| Mean (CI) | 48.9 (45.2, 52.5) | 46.5 (43.2, 49.9) | 47.7 (45.2, 50.2) |  |
| Median (Q1, Q3) | 49.3 (37.9, 59.7) | 46.7 (33.6, 59.8) | 52.9 (35.3, 59.7) |  |
| **Gender** |  |  |  | P>0.05 |
| Male | 49 (53.8%) | 48 (52.7%) | 97 (53.3%) |  |
| Female | 42 (46.2%) | 43 (47.2%) | 85 (46.7%) |  |

**Figure S1. Normality of age distribution before matching****Figure S2. Normality of age distribution after matching**
